# Life Span Extension by Calorie Restriction Depends on Rim15 and Transcription Factors Downstream of Ras/PKA, Tor, and Sch9

**DOI:** 10.1371/journal.pgen.0040013

**Published:** 2008-01-25

**Authors:** Min Wei, Paola Fabrizio, Jia Hu, Huanying Ge, Chao Cheng, Lei Li, Valter D Longo

**Affiliations:** 1 Andrus Gerontology Center, University of Southern California, Los Angeles, California, United States of America; 2 Department of Biological Sciences, University of Southern California, Los Angeles, California, United States of America; 3 Department of Computational and Molecular Biology, University of Southern California, Los Angeles, California, United States of America; Stanford University Medical Center, United States of America

## Abstract

Calorie restriction (CR), the only non-genetic intervention known to slow aging and extend life span in organisms ranging from yeast to mice, has been linked to the down-regulation of Tor, Akt, and Ras signaling. In this study, we demonstrate that the serine/threonine kinase Rim15 is required for yeast chronological life span extension caused by deficiencies in Ras2, Tor1, and Sch9, and by calorie restriction. Deletion of stress resistance transcription factors Gis1 and Msn2/4, which are positively regulated by Rim15, also caused a major although not complete reversion of the effect of calorie restriction on life span. The deletion of both *RAS2* and the *Akt* and S6 kinase homolog *SCH9* in combination with calorie restriction caused a remarkable 10-fold life span extension, which, surprisingly, was only partially reversed by the lack of Rim15*.* These results indicate that the Ras/cAMP/PKA/Rim15/Msn2/4 and the Tor/Sch9/Rim15/Gis1 pathways are major mediators of the calorie restriction-dependent stress resistance and life span extension, although additional mediators are involved. Notably, the anti-aging effect caused by the inactivation of both pathways is much more potent than that caused by CR.

## Author Summary

Reduction in calorie intake is a well-established intervention that extends the life span of a variety of biological model organisms studied. Calorie restriction also delays and attenuates age-related changes in primates, although its longevity-promoting effect has not been demonstrated. Here, we utilized a single cell organism, baker's yeast, to examine the role of evolutionarily conserved genes in life span regulation and their involvement in calorie restriction. The yeast mutants lacking Ras2, Tor1, or Sch9 are long-lived. The anti-aging effect observed in these mutants depends on the protein Rim15 and several key regulators of gene expression that are essential in inducing cellular protection under stress. The beneficial effects of calorie restriction are much smaller in yeast that are missing these proteins, indicating their essential role in promoting longevity. Our study also showed that by combining the genetic manipulation and calorie restriction intervention, yeast can reach a life span ten times that of those grown under standard conditions. This extreme longevity requires Rim15 and also depends on other yet-to-be identified mechanisms. Our findings provided new leads that may help to elucidate the mechanisms underlying the anti-aging effect of calorie restriction in mammals.

## Introduction

The effect of restricting calorie intake on life span extension has been known for more than 70 years [1,2]. Although many hypotheses on how calorie restriction (CR) modulates aging have been proposed, the underlying mechanism for CR is still elusive [3]. Evidence from genetic studies utilizing model organisms ranging from yeast to mammals points to an important role of nutrient-sensing/insulin/insulin growth factor I (IGF-I) pathways in life span modulation, suggesting a common evolutionary origin of aging regulation [4]. Furthermore, these signaling pathways have been implicated in mediating CR-induced life span extension in yeast, flies, and mice [4–6].

In yeast, the conserved Ras, Tor, and Sch9 signaling pathways integrate the nutrient and other environmental cues to regulate cell growth/division [7,8]. Deletion of *SCH9*, a homolog of mammalian *AKT* and *S6K* [9,10], enhances cellular protection against thermal and oxidative challenges, and extends yeast chronological life span (CLS, defined as the survival of non-dividing cells) as well as replicative life span (RLS, defined as the number of daughter cells produced by a mother cell) [11,12]. Similarly, the *RAS2*-null strain shows increased stress resistance and survival [13–15]. Recently, evidence has been presented that deficiency in TORC1 signaling also promotes longevity in both the replicative and chronological model systems [6,16,17].

Rim15 is a glucose-repressible protein kinase and a key integrator of signals transduced by the Sch9, Ras, and Tor pathways in response to nutrients [18–20]. Nutrient depletion activates Rim15, which in turn upregulates the expression of a variety of genes involved in G_0_ entry and stress response through the transcription factors Msn2/4 and Gis1 [21]. We have previously reported that life span extension associated with deficiencies in Sch9 and Ras2/cAMP/PKA is partially mediated by enhanced cellular protection against oxidative stress through the activation of *SOD2* [13]. Both the stress response element (STRE) and post-diauxic shift motif (PDS) are present in the promoter region of *SOD2*, suggesting the involvement of stress response transcription factors Msn2/4 and Gis1 [22,23]. In fact, deletion of *MSN2/4* in *ras2*Δ and of *RIM15* in *sch9*Δ mutants reverses or reduces life span extension [11]. Lack of Rim15 also abolishes the life span extension associated with a reduced activity of adenylate cyclase [13], which is found downstream of Ras2 in the Ras/PKA nutrient sensing pathway. Moreover, Msn2/4 and Rim15 are negatively regulated by the TORC1 signaling, which promotes the cytoplasmic retention of Msn2/4 and Rim15 through the interaction with the 14-3-3 protein BMH2 [24,25]. Genetic data also suggest that Tor inhibits protein phosphatase 2A-dependent nuclear accumulation of Msn2 in response to stresses [26].

CR delays aging and prolongs chronological and replicative life span in yeast [27–30]. For RLS studies, CR can be modeled by maintaining yeast cells on reduced glucose concentration but otherwise complete (rich) medium [28,29]. CR fails to further extend the RLS of either *sch9*Δ or *tor1*Δ mutants, indicating that down-regulation of the Tor and Sch9 pathways may mediate CR effect in dividing yeast [6]. In liquid culture, yeast cells growing in glucose containing medium release and accumulate ethanol, which promotes cell death in wild-type cells during chronological aging [30]. Switching non-dividing yeast cells from ethanol-containing medium to water, which models the extreme CR/starvation condition that yeast encounter in the wild, extends not only the mean life span of wild-type cells but also that of *sch9*Δ mutants, indicating the presence of additional mechanism(s) controlled by CR [27,30].

Here we present results showing that the serine/threonine kinase Rim15 and the downstream stress resistance transcription factors Msn2/4 and Gis1 are required for chronological life span extension in mutants with defects in Ras/cAMP/PKA or Tor/Sch9 signaling as well as in calorie restricted cells. In addition, we show that calorie restriction/starvation doubles the chronological life span of the extremely long-lived mutants lacking both *RAS2* and *SCH9*, and that this 10-fold life span extension is only partially dependent on Rim15. Our findings are consistent with the existence of a longevity regulatory network centered on the Ras/cAMP/PKA/Rim15/Msn2/4 and Tor/Sch9/Rim15/Gis1 pathways which play important roles in the mediation of CR-dependent stress resistance and life span extension. However, our results also indicate that mutations in Tor, Sch9, and Ras signaling in long-lived mutants do not recapitulate the full effect of CR, and both Rim15/Msn2/4/Gis1-dependent and -independent mechanisms are required to achieve maximum life span extension.

## Results

### Role of Rim15 and Gis1 in Regulating Yeast Chronological Life Span

**Figure 1:**
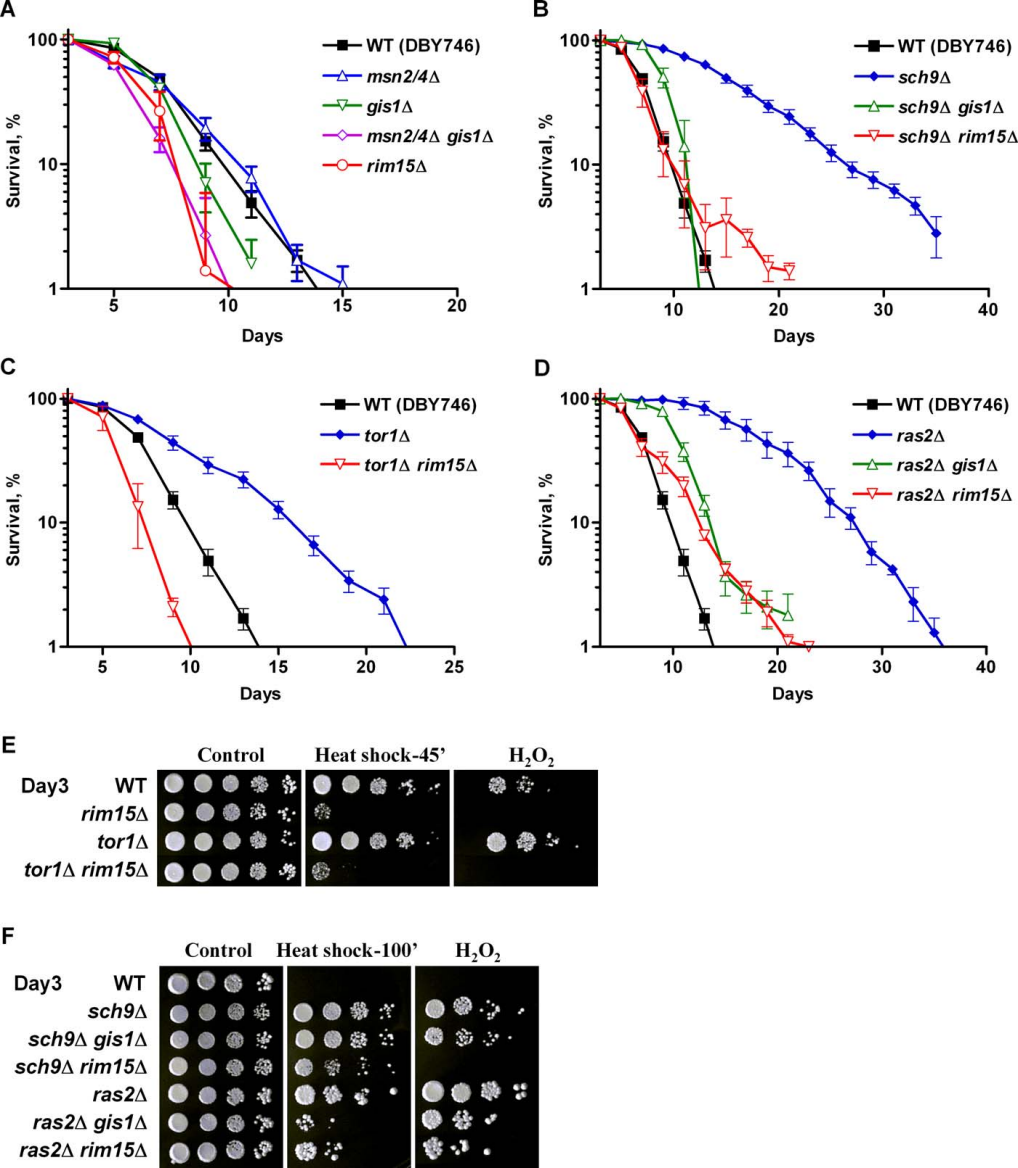
Rim15 Is Required for Chronological Life Span Extension and Cellular Protection (A) CLS of wild-type (DBY746) and mutants lacking Rim15 or downstream stress response transcription factors: Msn2/4 and/or Gis1. (B–D) Deletion of *RIM15* reverses life span extension associated with deficiencies in Sch9 (B), Tor1 (C), or Ras2 (D). Deletion of *GIS1* partially reverses CLS extension of *sch9*Δ (B) and *ras2*Δ (D) mutants. (E) Day 3 cells were subject to thermal (55 °C, 45 min) and oxidative stress (hydrogen peroxide, 100 mM, 60 min). Strains shown are wild-type, *rim15*Δ, *tor1*Δ, and *tor1*Δ *rim15*Δ. (F) Day 3 cells were subject to thermal (55 °C, 100 min) and oxidative stress (hydrogen peroxide, 150 mM, 60 min). Strains shown are wild-type, *sch9*Δ, *sch9*Δ *gis1*Δ, *sch9*Δ *rim15*Δ, *ras2*Δ, *ras2*Δ *gis1*Δ, and *ras2*Δ *rim15*Δ.

Previously, we have shown that deficiencies in Ras and Sch9 signaling pathways extend yeast chronological life span through, in part, the activation of the stress response transcription factors Msn2/4 and protein kinase Rim15, respectively [11,13]. Since Rim15 has also been shown as the integrating point of the Tor and Ras/PKA nutrient-sensing pathways and an important regulator for G_0_ entry [21,25,31], we examined its role in yeast chronological life span extension caused by mutations in *tor1*Δ and *ras2*Δ mutants. The mean life span of *rim15*Δ mutant was slightly reduced (12%) compared to that of wild-type (DBY746) (Figure 1A; Table S1). Deletion of *RIM15* abolished life span extension associated with deficiencies in Tor1, Ras2, or Sch9 (Figure 1C and 1D; Table S1), suggesting that the longevity regulatory network controlled by Tor, Sch9, and Ras converges on Rim15.

Activation of cellular protection mechanisms represents an important survival strategy in yeast [32]. We tested the role of Rim15 in cellular protection in *tor1*Δ, *sch9*Δ, and *ras2*Δ mutants. Cells lacking Rim15 were hypersensitive to thermal and oxidative challenges (Figure 1E). Deletion of *Rim15* not only abolished protection against hydrogen peroxide, and to a lesser extent to heat, in *sch9*Δ (Figure 1F), it also abolished any beneficial effect associated with attenuated Tor signaling (Figure 1E). However, Rim15-mediated stress resistance only accounted for part of the stress resistance phenotype observed in *ras2*Δ mutant (Figure 1F).

**Figure 2:**
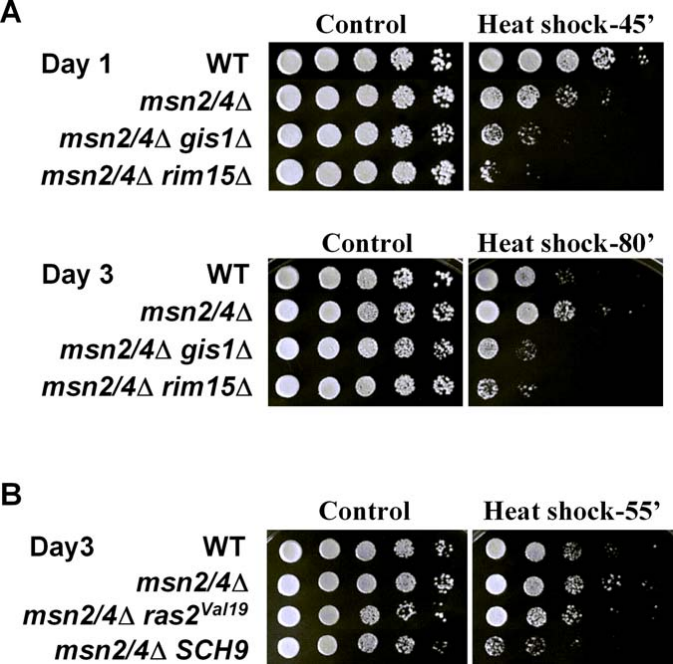
Thermal and Oxidative Stress Resistance of Cells Deficient of Stress Response Transcription Factors Msn2/4 and/or Gis1 (A) Day 1 and day 3 cells were exposed to heat stress (55 °C). Strains shown are wild-type (DBY746), *msn2*Δ *msn4*Δ, *msn2*Δ *msn4*Δ *gis1*Δ, and *msn2*Δ *msn4*Δ *rim15*Δ. (B) Day 3 cells were subject of heat stress (55 °C). Strains shown are wild-type, *msn2*Δ *msn4*Δ, and *msn2*Δ *msn4*Δ mutants overexpressing *ras2^val19^* or *SCH9*.

Rim15 activates Gis1, a transcription factor that binds to the PDS element (AWAGGGAT), and induces a variety of stress response genes when cells enter stationary phase [23]. To determine the contribution of Gis1 to chronological survival and cellular protection, we monitored CLS of the *gis1*Δ mutant as well as cells lacking *GIS1* in the long-lived genetic backgrounds. *gis1*Δ mutant had a mean life span similar to that of wild-type yeast (Figure 1A; Table S1). In contrast, the survival of the *msn2*Δ *msn4*Δ *gis1*Δ triple mutant was shorter than that of wild-type and resembled that of *rim15*Δ (Figure 1A; Table S1), in agreement with the gene expression profile data suggesting that Msn2/4 and Gis1 cooperatively mediate the Rim15 response to glucose limitation [19,21]. Deficiency in Gis1 almost completely abolished the mean life span of *sch9*Δ mutant (Figure 1B), in agreement with our earlier finding regarding the role of Rim15 in mediating the effect of *sch9*Δ mutation in stress resistance and life span [11]. In the *RAS2*-null background, the enhanced survival effect was not fully dependent on Gis1 (Figure 1D; Table S1). This observation may be explained by the fact that Msn2/4 play an important role in the life span extension associated with *ras2*Δ [13]. With respect to cellular protection, 1-d-old *msn2*Δ *msn4*Δ mutant was hypersensitive to both heat and oxidative stresses as expected (Figure 2A and unpublished data). At day 3, however, the mutant showed more than 10-fold increase in resistance to heat, but not to hydrogen peroxide (Figure 2A and unpublished data). This phenotype was not due to an adaptive mutagenesis, as the frequency of canavanine-resistant (*can^R^*) mutation did not differ significantly between *msn2*Δ *msn4*Δ mutant and that of wild-type (Figure S1). Furthermore, the day 3 heat resistant *msn2*Δ *msn4*Δ cells were still sensitive to stress challenges 1 d after being re-inoculated in fresh medium (unpublished data). We showed that this compensatory activation of additional cellular protection in *msn2*Δ *msn4*Δ mutant at day 3 was Rim15/Gis1-dependent since it was abolished by deletion of either *RIM15* or *GIS1* (Figure 2A). The enhanced thermal resistance of *msn2*Δ *msn4*Δ seen at day 3 was also abolished by the overexpression of Sch9 or, to a lesser extent, the constitutively active Ras2 (*ras2^val19^*), both of which inhibit Rim15/Gis1 (Figure 2B). These results depict a Ras-, Tor-, and Sch9-controlled longevity regulatory network with Rim15 in the center transducing the signals to activate stress response genes and positively regulating life span (Figure 5B). It is notable that the degree of dependence on stress response transcription factors downstream of Rim15 is quite different in *sch9*Δ and *ras2*Δ mutants, with the former depending primarily on Gis1 and the latter on both Msn2/4 and Gis1.

### Extreme CR/Starvation further Extends the Life Span of Mutants with Deficiencies in Tor, Sch9, and Ras/cAMP/PKA Signaling

**Figure 3:**
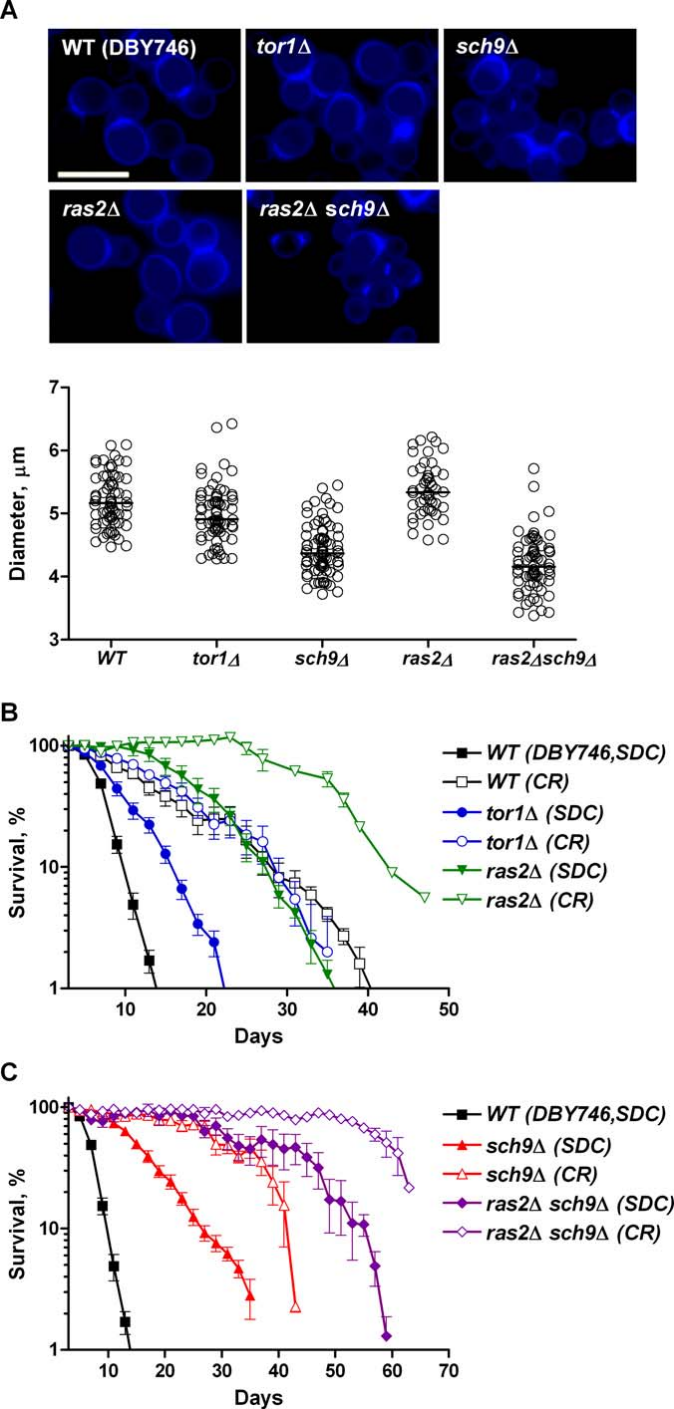
Cell Size Analysis and CLS under Extreme CR (A) Calcofluor staining of wild-type (DBY746), *tor1*Δ, *sch9*Δ, *ras2*Δ, and *ras2*Δ *sch9*Δ mutants. Bar, 10 μm. Scatter plot of cell diameters (the average of the long and short axes of the cell) with the bar indicating median. Fifty to 75 cells were measured per genotype. (B,C) Extreme CR, where day 3 SDC cultures were switched to water, further extends the life span of already long-lived genetic mutants. Strains shown are wild-type, *tor1*Δ *ras2*Δ, *sch9*Δ, and *ras2*Δ *sch9*Δ.

Tor, Sch9, and Ras/cAMP/PKA control a dynamic transcriptional network that regulates the balance between cell growth and division [7,8]. Whereas cells lacking *SCH9* are small in size (∼60% of that wild-type in volume) and display a slow growth phenotype, *tor1*Δ mutants are only slightly smaller than wild-type cells (∼86%) and grow at a normal rate (Figure 3A). This may be due to the fact that Tor2 can function, in redundancy to Tor1, in the TORC1 complex [33]. *RAS2*-null cells show a small increase in cell size (by 10% in volume) compared to wild-type. The combination of the *ras2*Δ and *sch9*Δ instead causes a further but small decrease in cell size (Figure 3A). Since all three mutants are long-lived despite differences in cell size and growth rate, it appears that chronological survival can be uncoupled from the signaling involved in regulating cell growth and size. This is particularly important considering that some of the longest-lived mutants in higher eukaryotes are dwarfs and it is not clear whether life span extension can be separated from dwarfism [4].

The down-regulation of the Sch9, Tor, or Ras pathways has been implicated in the mediation of the CR effect on longevity [6,28,34]. We have previously shown that extreme CR/starvation, in which stationary phase cells were switched to water, doubles the mean life span of wild-type yeast [30,35]. Furthermore, the life span of already long-lived *sch9*Δ is further extended by the removal of nutrients, suggesting that either the Sch9 pathway only partially mediates the CR effect or the mechanisms underlying CR are distinct from those triggered by the deletion of *SCH9* [30]. To understand the role of Tor, Ras, and Sch9 signaling in CR, we monitored the survival of *tor1*Δ, *ras2*Δ, and *ras2*Δ *sch9*Δ mutants in water. As observed with *sch9*Δ, starvation/extreme CR increased mean life span of both *TOR1*- and *RAS2*-null mutants (Figure 3B; Table S2). The mean (50% survival) and maximum (10% survival) life span was markedly increased in CR *ras2*Δ mutant compared to CR wild-type strain. This was not the case for *tor1*Δ mutant. Although CR further extended the life span of *tor1*Δ, there was only 18% increase in mean CLS, and no difference in maximum CLS compared to that of wild-type under extreme CR (Table S2). Considering that Rim15 is required for chronological survival extension for all three long-lived mutants, these results suggest that the Rim15-controlled Msn2/4 and Gis1 are differentially activated in *tor1*Δ, *sch9*Δ, and *ras2*Δ mutants. The fact that *ras2*Δ *sch9*Δ double mutant survive longer than either one of the single mutants (Figure 3C) supports this conclusion and suggests that the full beneficial effect of CR may be accounted by the combined effect of down-regulation of both Ras2 and Sch9 signaling. To our surprise, however, extreme CR extended the survival of *ras2*Δ *sch9*Δ double knockout mutant, which reached a mean life span of approximately 10-fold of that wild-type grown and incubated in standard glucose/ethanol medium (Figure 3C; Table S2). This suggests an additive effect between down-regulation of both the Ras/cAMP/PKA and Sch9 pathways and dietary interventions. Alternatively, Ras/cAMP/PKA signaling could be down-regulated further by the inactivation of Ras1 by CR. In fact, Ras1 and Ras2 play redundant roles in the regulation of the cAMP/PKA pathway although their expression profile is different. Unfortunately, the *ras1*Δ *ras2*Δ double mutant could not be tested because it is not viable.

### Rim15 and the Stress Response Transcription Factors Msn2, Msn4, and Gis1 Are Required for CR-induced Longevity Extension

**Figure 4:**
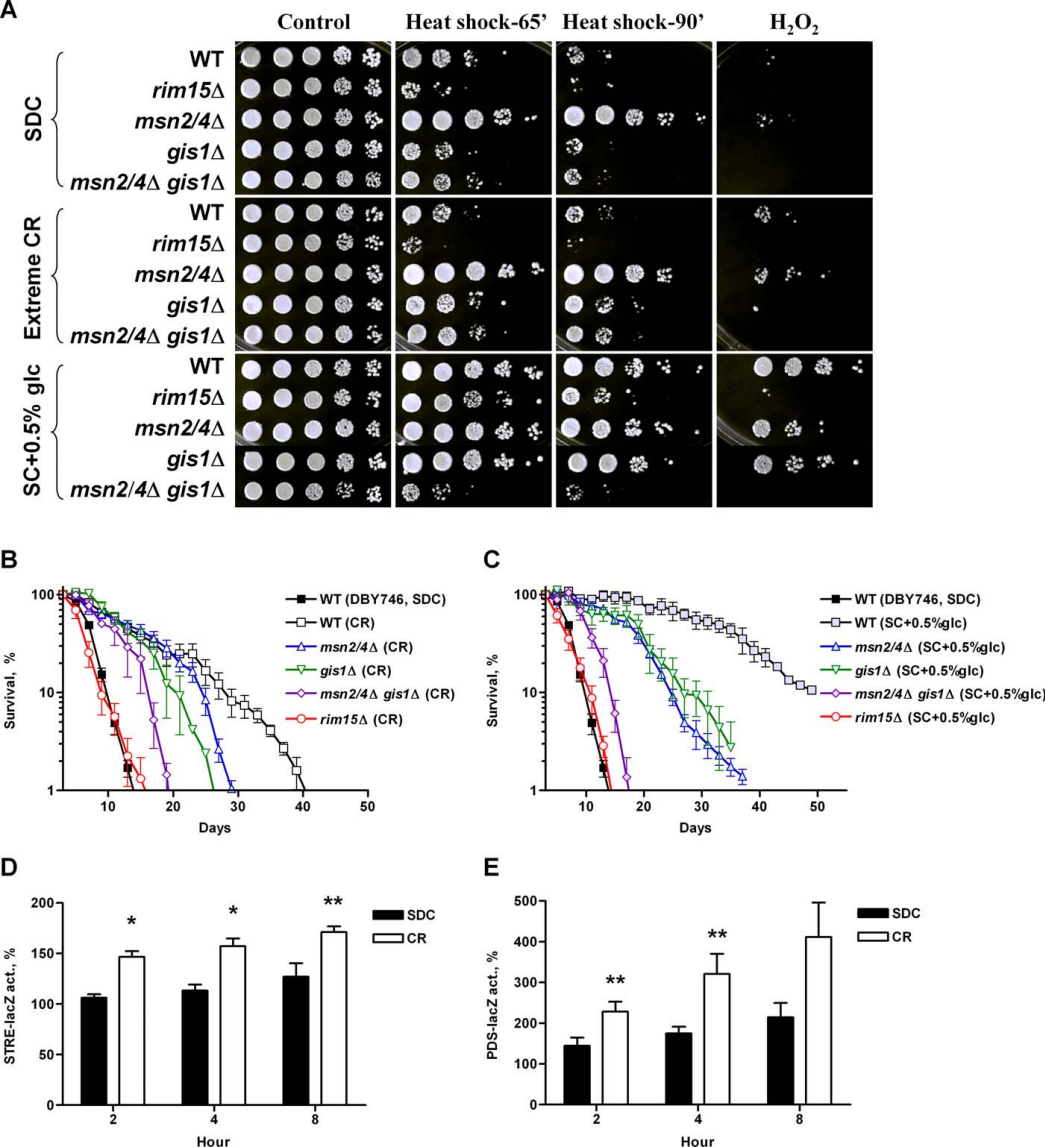
CR-Induced Cellular Protection and Life Span Extension Require Rim15 and Stress Response Transcription Factors (A) Effects of extreme CR/starvation and glucose reduction on cellular protection against thermal and oxidative stress. For extreme CR/starvation, cells from day 3 SDC cultures were switched to water. For glucose reduction, cells were grown in SC + 0.5% glucose medium. Stress resistance assay was performed at day 5 (48 h after switching to water). Cells were subject to heat shock (55 °C) or H_2_O_2_ (176 mM, 60 min) stress. (B) CLS under extreme CR/starvation. Strains shown are wild-type (DBY746), *msn2*Δ *msn4*Δ, *gis1*Δ, *msn2*Δ *msn4*Δ *gis1*Δ, and *rim15*Δ. (C) CLS under CR modeled by glucose reduction (SC + 0.5% glucose). Strains shown are wild-type, *msn2*Δ *msn4*Δ, *gis1*Δ, *msn2*Δ *msn4*Δ *gis1*Δ, and *rim15*Δ. (D,E) Day 1 SDC wild-type cells were switched to water. The STRE-lacZ (D) and PDS-lacZ (E) activities were measured 2 h, 4 h, and 8 h after the initiation of CR and shown as the percentage of time 0. Data shown are mean ± standard error of the mean of four independent samples assayed. *, *p* < 0.01; **, *p* < 0.05, two-tailed t-test, CR versus SDC.

To elucidate the roles of Rim15 and its downstream transcription factors in CR, we monitored the stress resistance and chronological survival of cells lacking Rim15, Gis1, and/or Msn2/4 incubated in water. This extreme CR treatment caused a ∼10-fold increase in oxidative defense in wild-type as well as in mutants lacking Msn2/4 (Figure 4A). On the other hand, the *gis1*Δ, *msn2*Δ *msn4*Δ *gis1*Δ, and *rim15*Δ mutations prevented the enhancement in resistance to stress (Figure 4A). The commonly used CR protocol in S. cerevisiae involves a reduction in glucose concentration from 2% to either 0.5% or 0.05%, which has been shown to extend both the replicative and chronological life span [28,29,36–38]. In addition to the switch to water, we also tested the effect of the calorie restriction by reducing the glucose concentration in the growth medium from 2% to 0.5%. This CR intervention led to an even higher increase in the resistance to both heat shock and oxidative stress (Figure 4A). These effects of calorie restriction were also completely reversed by the lack of Rim15 or all three stress resistance transcription factors *MSN2*, *MSN4* and *GIS1*, but not by the lack of either Msn2/4 or Gis1 alone (Figure 4A).

Under the extreme CR condition, mean life span of the *msn2*Δ *msn4*Δ and *gis1*Δ did not differ significantly from that of wild-type, whereas a ∼25% reduction in maximum life span (measured as the age when 10% of the cells were still alive) was observed in *GIS1*-null mutant (Figure 4B; Table S2). Lack of all three stress response transcription factors led to a 50% reduction of maximum life span compared to wild-type (Figure 4B). By contrast, extreme CR/starvation failed to extend the longevity of Rim15-null mutant (Figure 4B). The results obtained under glucose reduction CR (0.5% glucose) were very similar to those under extreme CR with the exception that wild-type cells achieved a mean life span of 31 d instead of 12 d, and the deletion of *MSN2/4* had a more marked negative effect on this CR-dependent life span extension (Figure 4C). Taken together, these data suggest that the serine/threonine kinase Rim15 plays a central role in mediating the effect of CR on stress resistance and life span extension by positively regulating the activities of stress resistance transcription factors Msn2/4 and Gis1.

### STRE- and PDS-Dependent Gene Expression during Extreme CR

Activation of Msn2/4 and Gis1 leads to the expression of variety of stress response genes with STRE and PDS elements in their promoters. We employed the STRE- and PDS-driven reporter gene assay to examine the gene expression changes under extreme CR condition. One-day-old wild type cells carrying either STRE- or PDS-driven lacZ reporter gene were switched to water. Significant increase in both STRE- and PDS-driven transactivation was observed 2 h after the initiation of CR compared to cells maintained in SDC medium (Figure 4D and 4E). PDS-dependent transactivation increased by 90%, whereas STRE activation increased by 40%, under the extreme CR condition by 8 h. This observation is in agreement with our survival data that Gis1 plays a more important role in extreme CR-induced longevity extension (Figure 4B).

The statistical analysis of data derived from genome-wide motif prediction and global expression profiles provides a powerful tool to infer transcriptional regulation in the cell [39]. We have previously reported that there is significant enrichment of STRE and PDS elements in the promoter regions of the genes upregulated in *sch9*Δ mutant compared to wild-type under normal culture condition (SDC) [40]. Here, we analyzed the expression of genes containing STRE (Msn2/4) or PDS (Gis1) elements in their promoter under extreme CR (switching to water). Our data did not indicate an enrichment of either STRE or PDS element in genes upregulated under CR (either 24 or 48 h) in wild-type cells (Table 1). This is probably due to the fact that CR induced a significant but small increase (40% to 90%) in transactivation of Msn2/4 and Gis1 (Figure 4D), which could not be detected in the analysis of array data which was performed at a cutoff of 1.7-fold (CR versus SDC). However, CR (water) did cause a significant increase in the expression of STRE- and PDS-containing genes in the *sch9*Δ mutant (Table 1). These findings are consistent with the fact that CR further extends the life span of *sch9*Δ mutant, and support the notion that pathways responsible for cellular protection and life span extension in long-lived genetic mutant and in CR-treated cells are overlapping, although their levels of activation are not identical.

**Table 1:**
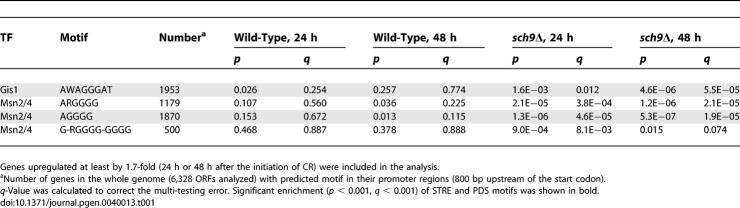
Significance of STRE and PDS Enrichment in Genes Upregulated under Extreme CR

### Maximum Life Span Extension Requires Rim15-Independent Signaling

**Figure 5:**
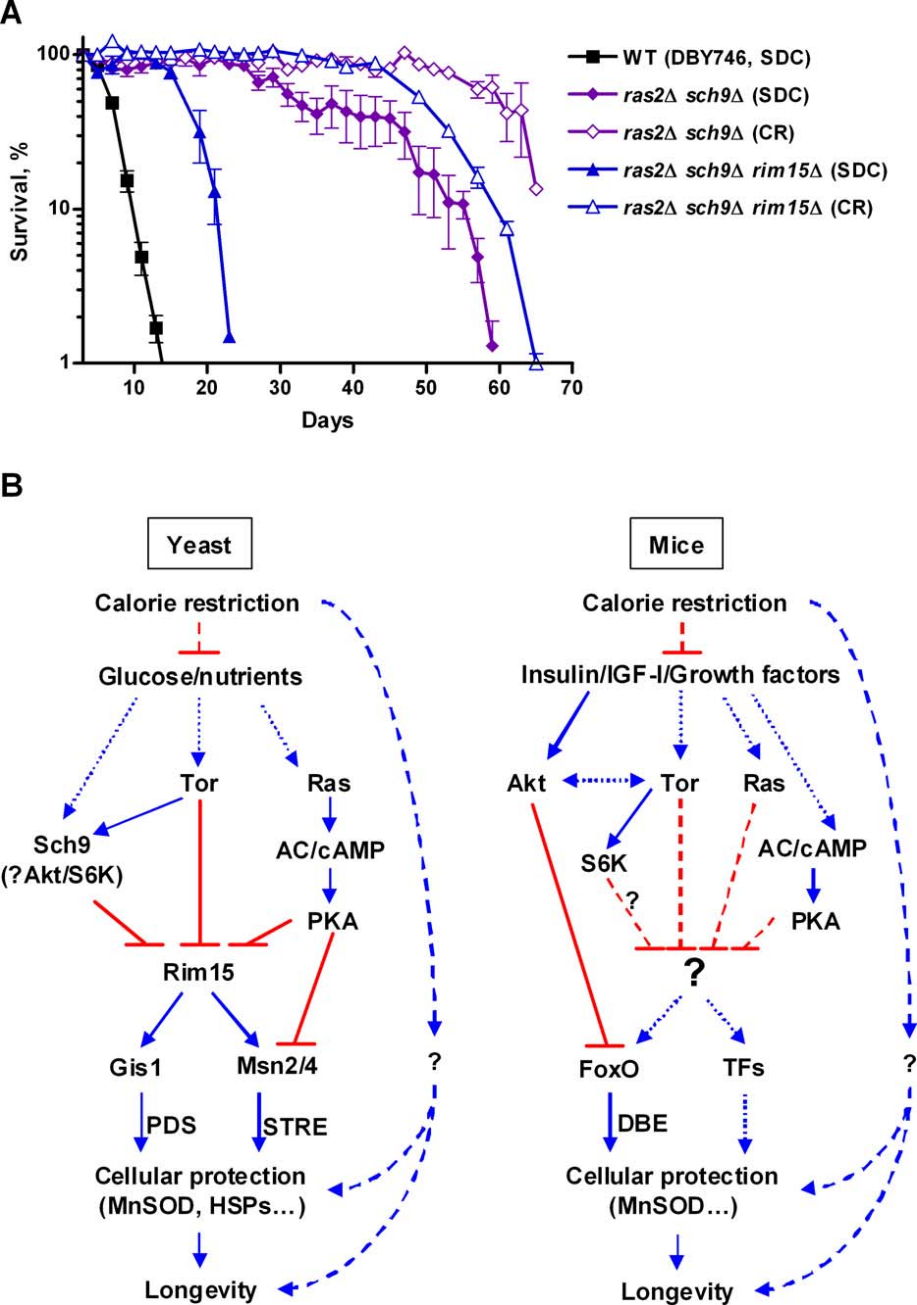
Rim15-independent Mechanism(s) Is Required for Maximal Life Span Extension (A) CLS under extreme CR. Strains shown are wild-type (DBY746), *ras2*Δ *sch9*Δ, and *ras2*Δ *sch9*Δ *rim15*Δ in standard SDC and under extreme CR. (B) Longevity regulatory pathways. In yeast, nutrient-sensing pathways controlled by Sch9, Tor, and Ras converge on the protein kinase Rim15. In turn, the stress response transcription factors Msn2, Msn4, and Gis1 transactivate stress response genes and enhance cellular protection, which leads to life span extension. Although a major portion of the effect of CR on longevity appears to be mediated by the down-regulation of the Ras and Tor-Sch9 pathways and consequent activation of the Rim15-controlled Msn2/4 and Gis1 protection system, additional mediators are involved. In mice, the partially conserved insulin/IGF-I-like pathways negatively regulate the FoxO family transcription factors through the Sch9 homolog Akt. Ras and Tor also function downstream of IGF-I, although their role in the regulation of stress resistance and aging are poorly understood. Mice deficient in type 5 adenylyl cyclase are long-lived and stress resistant analogously to the adenylate cyclase deficient yeast. However, the mediators of life-span extension in GH-, IGF-I-, or AC-deficient as well as CR mice have yet to be identified.

To determine whether the life span regulatory effects caused by deficiencies in the Ras/cAMP/PKA and Sch9 pathways were additive, we studied the *ras2*Δ *sch9*Δ double mutants. Cells lacking both *RAS2* and *SCH9* showed a mean CLS of 35 d, which is more than 5-fold that of wild-type cells (Figure 5A; Table S1). Surprisingly, extreme CR/starvation caused an additional doubling of the life span of the *ras2*Δ *sch9*Δ (10-fold that of wild-type in glucose/ethanol medium) (Figure 5A; Table S1). In view of the important role of Rim15 in life span extension in both the long-lived *ras2*Δ and *sch9*Δ mutants as well as in the CR-dependent effects, we examined the role of *RIM15* in the longevity regulation by *ras2*Δ *sch9*Δ. Lack of Rim15 only partially reversed the life span extension associated with deficiencies in both Ras2 and Sch9 (from more than 5-fold to 2.5-fold, Figure 5A; Table S2). The reversion was even less prominent in mutants under extreme CR, where the 10-fold life span extension was reduced to 7.5-fold (Figure 5A; Table S2). These data indicate that Rim15-independent pro-longevity mechanisms are activated in mutants lacking Ras2 and Sch9 signaling and that their beneficial effects are further potentiated by the extreme CR intervention.

## Discussion

Model organisms including yeast, worms, flies, and mice have been studied extensively to understand the mechanisms of aging. Here we present genetic evidence that both CR and evolutionarily conserved signal transduction proteins implicated in life span regulation, including Ras, Tor, and Sch9, require the serine/threonine protein kinase Rim15 and the downstream stress resistance transcription factors Msn2/4 and Gis1 to extend life span. However, additional factors appear to be involved in the remarkable 10-fold life span extension observed in calorie restricted *ras2*Δ *sch9*Δ mutants.

We have previously reported that life span extension in *SCH9*-null and adenylate cyclase deficient mutants depends on Rim15 [11,13]. Here we show that deletion of *RIM15* also completely abolished the life span extension as well as the stress resistance phenotype caused by the deficiencies in Ras or Tor signaling. The activity of Rim15 has been shown to involve stress response transcription factors Msn2, Msn4, and Gis1 [19,20,21]. Deficiency in Gis1 led to a reversion of life span extension of the *sch9*Δ and, to a lesser extent, *ras2*Δ mutants. These data are consistent with the existence of at least two major life span regulatory pathways controlled by Ras/cAMP/PKA and Tor/Sch9, both of which converge on Rim15. The present data also point to an important role of stress response transcription factors controlled by Rim15, Msn2/4, and Gis1, in mediating the pro-longevity effect in all long-lived genetic mutants with deficiencies in nutrient sensing pathways (Figure 5B).

To study the CR effect on yeast chronological survival, we took two different approaches, starvation and glucose reduction. The first one models the extreme condition that yeast encounter in the wild during complete starvation periods. The extreme CR may be considered as a dietary restriction since all the nutrients in addition to calories are removed from the culture. The reduction of glucose from 2% to 0.5% instead is the calorie restriction regimen commonly used in RLS and CLS studies [28,29,36–38]. Both CR interventions increased cellular protection and extended chronological survival of wild-type cells, with glucose reduction showing a more powerful effect. The difference may be explained, at least in part, by the onset of CR. Unlike the starvation paradigm, in which CR was initiated after cells had entered stationary phase, cells growing in low glucose medium were exposed to CR from the very beginning. In agreement with the hormesis hypothesis of CR [34,41], it is possible that the mild stress imposed by CR early in life leads to an adaptive redirection of energy and resource from growth to survival. Another possibility is that the early reduction of glucose concentration causes changes in gene expression that affect stress resistance and survival at later stages. Others have shown that CR failed to increase the replicative life span of Tor1- or Sch9-deficient mutants [6]. Our results show that the CR effect requires Tor/Sch9-controlled protein kinase Rim15 and its downstream stress response transcription factors. However, extreme CR/starvation further extended the chronological life span of the already long-lived *tor1*Δ, *sch9*Δ, and *ras2*Δ mutants. This difference may be the result of the very different paradigms to study aging: RLS measures the budding potential of a mother cell, whereas the CLS measures the survival of non-dividing cells. It may also be due to the CR paradigms utilized, i.e., glucose reduction but constant exposure to 0.5% glucose and other nutrients (RLS) versus starvation in water (CLS). The amino acids or other nutrients still present in the RLS paradigm may block the effect of starvation/CR on the Ras pathway and other stress resistance transcription factors. In fact, RLS extension was also achieved by decreasing the amino acid content of the medium [29]. In our CLS starvation paradigm instead, all nutrients that may contribute to the activation of pro-aging pathways are removed.

The CR effect was completely reversed in cells lacking the protein kinase Rim15 but not in the *msn2*Δ *msn4*Δ *gis1*Δ triple mutants, suggesting the presence of additional Rim15-dependent transcriptional factor(s) or signaling component(s) yet to be identified. Forkhead family transcription factors are evolutionarily conserved from yeast to mammals and have been implicated as mediators of insulin/IGF-I/Akt signaling pathway in the regulation of anti-aging genes in worms, flies, and mammals [42]. PHA-4, a forkhead transcription factor orthologous to the mammalian Foxa, has been shown to mediate the dietary restriction effect in C. elegans [43]. Results from our preliminary studies on the single deletion mutants of the four known forkhead TFs in S. cerevisiae (i.e., Fhl1, Fkh1, Fkh2, and Hcm1) are not consistent with a major life span regulatory role of these proteins (unpublished data). Instead, data presented in this study point to zinc finger transcription factors Msn2/4 and Gis1 as key components of the CR-dependent pro-longevity pathway. Based on the database search, the immediate early genes of the Egr-1 family of C2H2-type zinc-finger proteins show the highest score of homology to Msn2/4 [44]. The Egr-1 family TFs have been implicated in a variety of cellular processes including differentiation, mitogenesis, DNA repair, senescence, and apoptosis [45,46]. Mammalian Sp1- and Kruppel-like transcription factors are among the candidates homologous to Gis1. They are involved in insulin- and TGFβ-signaling. Interestingly, Gis1 also contains a jumonji domain, which is first described as a bipartite protein domain present in many eukaryotic transcription factors [47]. Recent evidence from several organisms has shown that a number of jmjC domain-containing proteins are histone demethylases, suggesting a role of Jumonji-domain–containing protein in chromatin remodeling [48]. Interestingly, the DNA binding activities of Egr-1, Sp1, and other zinc-finger TFs are sensitive to cellular redox state, and their dysfunction during aging may lead to age-associated pathophysiology [49–52]. While the existence of conserved domains in these yeast proteins is encouraging, it is still premature to speculate about their mammalian counterparts.

Although the protein kinase Rim15 is required for life span extension in Ras2, Tor1, and Sch9-deficient mutants as well as in yeast under CR, our results indicate that pathways responsible for enhancing stress protection and life span extension in nutrient sensing-impaired genetic mutants and in cells under CR are not identical. On the one hand, the “full” activation of Rim15 and its downstream transcription factors, Msn2/4 and Gis1, are required for the maximum life span extension, as the pro-longevity effects of *ras2*Δ, *sch9*Δ, and CR are additive (Figures 3B and 3C, and 5A). On the other hand, Rim15 only accounts for part of the beneficial effect for *ras2*Δ *sch9*Δ mutant under CR, implicating the involvement of additional pro-survival mechanism(s) independent of the Rim15-centered nutrient-sensing pathways (Figure 5A). Similar observations were also made in other model systems: CR can further increase the life span of the already long-lived Ames dwarf mice [53]; and it further extends the life span of insulin/IGF-I signaling-impaired *chico* flies [5].

We and others had shown that the down-regulation of Ras/cAMP/PKA signaling extends the yeast chronological and replicative life span [11,13,14,28]. However, the mammalian cAMP/PKA was only very recently implicated in the regulation of longevity in mice. The type 5 adenylyl cyclase knockout (AC5-KO) mice live 30% longer than their wild-type littermates [54]. CA5-KO mice do not show dwarfism, although they weigh slightly less than age-matched controls at 28 months. Similarly to mutants lacking Ras2 or with a reduced adenylate cyclase activity, mouse cells with CA5 disruption show enhanced resistance to oxidative stress, which may be mediated by the upregulation of MnSOD [13,54]. Interestingly, the CA5-KO mice have lower growth hormone level [54], suggesting an attenuated GH/insulin/Akt signaling in these mice. In view of our yeast data showing that CR in combination with the down-regulation of the Ras/cAMP/PKA and Sch9 pathways reached a 10-fold life span increase, it will be interesting to determine the interaction between the insulin/Akt and Ras/cAMP/PKA pathways as well as their combined effect with CR in regulating life span in mammals. Considering the fact that Ras and Sch9 signaling pathways are partially conserved from yeast to mammal (Figure 5B), it will also be important to explore the possibility that potential orthologs of Rim15 and of Msn2/4 and Gis1 may modulate aging in high eukaryotes.

## Materials and Methods

### Yeast strains.

All strains used in this study are derivatives of DBY746 (*MAT*α *leu2–3, 112, his3*Δ*, trp1–289, ura3–52, GAL^+^*). Knockout strains were generated by one-step gene replacement as described previously [55]. Strains overexpressing *SCH9* or *ras2^val19^* were generated by transforming cells with plasmids pHA_3_-*SCH9* (a gift from Dr. Morano), or pMW101 (plasmid RS416 carrying *Cla*I-*ras2^val19^*-*Hind*III fragment form pMF100, a gift from Dr. Broach), respectively. For strains used in STRE- and PDS-lacZ reporter gene assay, the plasmid pCDV454 containing LacZ reporter under the control of a 37 bp *SSA3*-PDS region (−206 to −170) [23] or the plasmid pMM2 containing four tandem repeats of STRE motif from the *HSP12* sequence (−221 to −241) [56], was integrated into the *URA3* locus of wild-type cells. The transcriptional specificity of these reporter genes were confirmed in the *msn2*Δ *msn4*Δ and *gis1*Δ background, respectively (unpublished data).

### Growth conditions and chronological life span assay.

Yeast cells were grown in SDC supplemented with a 4-fold excess of the tryptophan, leucine, uracil, and histidine to avoid possible artifacts due to auxotrophic deficiencies of the strains. Yeast chronological life span was measured as previously described [11,57]. Briefly, overnight SDC culture was diluted (1:200) in to fresh SDC medium to a final volume of 10 ml (with flask to culture volume of 5:1) and were maintained at 30 °C with shaking (200 rpm) to ensure proper aeration. This time point was considered day 0. Every 2 d, aliquots from the culture were properly diluted and plated on to YPD plates. The YPD plates were incubated at 30 °C for 2 d to 3 d, and viability was accessed by Colony Forming Units (CFUs). Viability at day 3, when the yeast had reached the stationary phase, was considered to be the initial survival (100%). Mean and maximum life span (10% survival) was calculated from curve fitting (one phase exponential decay) of the survival data (form pair matched, pooled experiments) with the statistical software Prism (GraphPad Software). For extreme CR/starvation, cells from 3-d-old SDC culture were washed three times with sterile distilled water, and resuspended in water. Water cultures were maintained at 30 °C with shaking. Every 2 d to 4 d, cells from the water cultures were washed to remove nutrients released from dead cells. For CR modeled by glucose reduction, overnight SDC culture was diluted (1:200) into fresh SC medium supplemented with 0.5% glucose. It is notable that the glucose reduction model employed here is different from that in replicative life span (RLS) studies. For RLS analysis, cells are maintained on reduced glucose but otherwise complete (rich) medium. In liquid chronological cultures, extracellular glucose was exhausted by day 1 in SC + 0.5% glucose as well as standard SDC cultures (unpublished data). Unlike the extreme low glucose cultures (SC + 0.05% glucose) which reached saturation density of only a quarter of that standard SDC ones, there was no difference in saturation density between cultures with 2% and 0.5% glucose, suggesting 0.5% glucose is not a limiting factor on cell growth/division (unpublished data).

### Stress resistance assay.

Heat shock resistance was measured by spotting serial dilutions (10-fold dilution started at OD_600_ of 10) of cells removed from SDC cultures onto YPD plates and incubating at either 55 °C (heat-shocked) or 30 °C (control) for 45 min to 150 min. After the heat-shock, plates were transferred to 30 °C and incubated for 2 d to 3 d. For oxidative stress resistance assays, cells were diluted to an OD_600_ of 1 in K-phosphate buffer, pH6.0, and treated with 100 mM to 200 mM of hydrogen peroxide for 60 min. Serially diluted (10-fold) control or treated cells were spotted onto YPD plates and incubated at 30 °C for 2 d to 3 d.

### Cell size analysis.

Day1 SDC cultures were mixed with equal volume of 2× calcofluor (75 ng/ml in PBS, Molecular Probe). After 10 min incubation at room temperature in the dark, cells were washed once with PBS. Images were captured with a Leica fluorescence microscope. Diameter of the cell was measured using ImageJ (http://rsb.info.nih.gov/ij/). Cells were measured at long and short (perpendicular) axes. Diameter was expressed as the average of the long and short axes of the cell. 50 to 80 cells per genotype were measured.

### Reporter gene assay.

Day 1 wild-type cells carrying the STRE- or PDS-lacZ reporter gene (grown in SDC) were split into two portions. One was washed three times with sterile water and resuspended in water; the other was maintained in the original SDC medium. Cells were collected at 2 h, 4 h, and 8 h after the initiation of CR. Cell pellet from 1 ml of culture was lysed with Y-PER (Pierce) according to manufacturer's protocol. The protein concentration of the lysate was assayed with a BCA kit (Pierce). 55 μl of lysate was mixed with 85 μl of substrate solution (1.1mg/ml ONPG in 60 mM Na_2_HPO_4_, 40 mM NaH_2_PO_4_, 10 mM KCl, 1 mM MgSO_4_, 50 mM 2-mercaptoethanol, pH7.0). Absorbance at 420 nm was read every 5 min until 30 min after the initiation of reaction. LacZ activities were determined by fitting the A_420_/time data to that of serial diluted recombinant β-galactosidase (Promega). LacZ activity was normalized to the total protein in the lysate.

### Microarray analysis and motif enrichment test.

A slight modified CR protocol was adopted, where 1.5-d-old cells were washed three times and incubated in water. 24 h and 48 h later, cells were collected for RNA extraction. These time points (to obtain RNA samples at day 2.5 and day 3.5) were selected to avoid the general decrease in metabolism and consequently in gene expression that normally occurs at older ages (day 4 to day 5) [57]. The cRNA generated from these samples was hybridized to Affymetrix GeneChip Yeast 2.0 array to obtain the measurement of gene expression. The “Invariant Set” approach was used for normalization at the probe level, and the “Model based” method to summarize and obtain expression for each probe set [58]. A detailed method for motif prediction and motif enrichment test has been described previously [40]. Briefly, for a given gene, if one or more binding sites of a transcription factor (TF) binding motif were found within the 800 bp region upstream of the start codon, it was defined as the target gene of that TF. A total of 51 motifs that can be associated with known TFs were used for motif prediction in all known yeast ORFs [39]. The cut-off value of motif matching score was set to 0.6. The hypergometric test was employed to determine whether there was an enrichment of any motif in CR-induced genes (upregulated more than 1.7-fold). Finally, we calculated the q-values for each test to correct the multiple testing errors using the “qvalue” package [59].

## Supporting Information

Figure S1 Mutation Frequency (Canavine Resistance Mutantation, *can^R^*) during Chronological Survival In parallel to chronological life span assay (in SDC medium), an aliquot of culture was harvested. Cells were washed once with sterile water and plated onto selective medium (SDC minus Arginine, supplemented with 60 mg/l L-canavanine sulfate). *can^R^* mutant colonies were counted after 2-d incubation at 30 °C. Strains shown are wild-type (DBY746), *msn2*Δ *msn4*Δ, *gis1*Δ, *msn2*Δ *msn4*Δ *gis1*Δ, and *rim15*Δ. At least four cultures for each genotype were analyzed. Data are presented as mean ± standard error of the mean.
(30 KB DOC)

Table S1 Chronological Life Span (52 KB DOC)

Table S2 Chronological Life Span under Calorie Restriction (52 KB DOC)

### Accession Numbers

Genes examined in this study from the Saccharomyces Genome Database (http://db.yeastgenome.org/) are as follows: *SCH9*, (YHR205W); *RAS2* (YNL098C); *TOR1* (YJR066W); *RIM15* (YFL033C); *MSN2* (YMR037C); *MSN4* (YKL062W); and *GIS1* (YDR096W).
