# Supplementary material for: Life Span Extension by Calorie Restriction Depends on Rim15 and Transcription Factors Downstream of Ras/PKA, Tor, and Sch9
Source: PLoS Genet. 2008 Jan 25;4(1):e13. doi: 10.1371/journal.pgen.0040013 (PMC2213705; doi:10.1371/journal.pgen.0040013)
Supplement: Figure S1 — In parallel to chronological life span assay (in SDC medium), an aliquot of culture was harvested. Cells were washed once with sterile water and plated onto selective medium (SDC minus Arginine, supplemented with 60 mg/l L-canavanine sulfate). canR mutant colonies were counted after 2-d incubation at 30 °C. Strains shown are wild-type (DBY746), msn2Δ msn4Δ, gis1Δ, msn2Δ msn4Δ gis1Δ, and rim15Δ. At least four cultures for each genotype were analyzed. Data are presented as mean ± standard error of the mean. (30 KB DOC) [file pgen.0040013.sg001.doc]

**Figure S1.**

**Figure S1.** Mutation frequency (canavine resistance mutantation, *can^R^*) during chronological survival. In parallel of chronological life span assay (in SDC medium), an aliquot of culture was harvested. Cells were washed once with sterile water and plated onto selective medium (SDC minus Arginine, supplemented with 60 mg/l L-canavanine sulfate). *can^R^* mutant colonies were counted after 2-day incubation at 30°C. Strains shown are wild type (DBY746), *msn2*Δ *msn4*Δ, *gis1*Δ, *msn2*Δ *msn4*Δ *gis1*Δ, and *rim15*Δ. At least four cultures for each genotype were analyzed. Data are presented as mean±SEM.
