# Supplementary material for: Life Span Extension by Calorie Restriction Depends on Rim15 and Transcription Factors Downstream of Ras/PKA, Tor, and Sch9
Source: PLoS Genet. 2008 Jan 25;4(1):e13. doi: 10.1371/journal.pgen.0040013 (PMC2213705; doi:10.1371/journal.pgen.0040013)
Supplement: Table S1 — (52 KB DOC) [file pgen.0040013.st001.doc]

**Table S1.** Chronological life span^1^.

|  | **Mean CLS** | | **10% survival** | |  |  |  |
| --- | --- | --- | --- | --- | --- | --- | --- |
|  | **days** | **%^2^** | **days** | **%^2^** | **n^3^** | ***p*^4^** | ***p^5^*** |
| WT (DBY746) | 6.5 | 100 | 10.9 | 100 | 61 |  |  |
| *msn2/4∆* | 6.1 | 94 | 11.3 | 103 | 13 |  |  |
| *gis1∆* | 6.7 | 103 | 9.7 | 88 | 8 |  |  |
| *msn2/4∆ gis1∆* | 5.3 | 82 | 8.8 | 80 | 4 |  |  |
| *rim15∆* | 5.7 | 88 | 8.9 | 81 | 10 |  |  |
| *sch9∆* | 15.3 | 235 | 27.3 | 249 | 37 | *p*<0.001 |  |
| *sch9∆ gis1∆* | 8.9 | 138 | 12.7 | 116 | 6 |  | *p*<0.05 |
| *sch9∆ rim15∆* | 6.2 | 96 | 11.6 | 106 | 6 |  | *p*<0.001 |
| *tor1∆* | 8.6 | 132 | 16.3 | 149 | 31 | *p*<0.001* |  |
| *tor1∆ rim15∆* | 5.4 | 83 | 8.6 | 78 | 4 |  | *p*<0.01** |
| *ras2∆* | 18.8 | 290 | 29.8 | 272 | 15 | *p*<0.001 |  |
| *ras2∆ gis1∆* | 10.3 | 158 | 15.9 | 146 | 6 |  | *p*<0.01 |
| *ras2∆ rim15∆* | 6.7 | 103 | 13.7 | 125 | 4 |  | *p*<0.001 |
| *ras2*Δ *sch9*Δ | 35.4 | 545 | 60.1 | 549 | 9 | *p*<0.001 |  |
| *ras2*Δ *sch9*Δ *rim15*Δ | 16.8 | 258 | 25.6 | 234 | 3 | *p*<0.001 | *p*<0.001 |

^1^ Data presented were calculated from pair matched, pooled experiments. ^2^ Percent of wild type (DBY746) in SDC. ^3^ “n” indicates the number of cultures analyzed. ^4^ *p*-value for mean CLS of mutants compared to that of wild type, ANOVA, Tukey's Multiple Comparison Test, except * *tor1*Δ *vs.* wild type, unpaired t-test, two-tailed. ^5^ *p*-value for mean CLS comparison, ANOVA, Tukey's Multiple Comparison Test, *p*-value shown are *sch9*Δ *gis1*Δ *vs.* *sch9*Δ; *sch9*Δ *rim15*Δ *vs.* *sch9*Δ; *ras2*Δ *gis1*Δ *vs.* *ras2*Δ; *ras2*Δ *rim15*Δ *vs.* *ras2*Δ; *ras2*Δ *sch9*Δ *rim15*Δ *vs.* *ras2*Δ *sch9*Δ, except ** *tor1*Δ *rim15*Δ *vs.* *tor1*Δ, unpaired t-test, two-tailed.
