# Supplementary material for: Life Span Extension by Calorie Restriction Depends on Rim15 and Transcription Factors Downstream of Ras/PKA, Tor, and Sch9
Source: PLoS Genet. 2008 Jan 25;4(1):e13. doi: 10.1371/journal.pgen.0040013 (PMC2213705; doi:10.1371/journal.pgen.0040013)
Supplement: Table S2 — (52 KB DOC) [file pgen.0040013.st002.doc]

**Table S2.** Chronological life span under calorie restriction^1^.

|  | **Mean CLS** | | **10% survival** | |  |  |  |
| --- | --- | --- | --- | --- | --- | --- | --- |
|  | **days** | **%^2^** | **days** | **%^2^** | **n^3^** | ***p*^4^** | ***p^5^*** |
| ***Extreme CR/Starvation*** | | | | | | | |
| WT (DBY746) | 12.7 | 195 | 28.6 | 261 | 22 |  | *p*<0.001 |
| *msn2/4∆* | 12.7 | 195 | 25.1 | 229 | 11 |  | *p*<0.001 |
| *gis1∆* | 12.5 | 192 | 21.3 | 195 | 4 |  | *p*<0.05 |
| *msn2/4∆ gis1∆* | 10.1 | 155 | 17.0 | 155 | 3 |  |  |
| *rim15∆* | 5.5 | 85 | 10.1 | 92 | 5 | *p*<0.001 |  |
| *tor1∆* | 15.0 | 230 | 28.2 | 258 | 12 |  | *p*<0.001 |
| *sch9∆* | 30.0 | 462 | n/a | n/a | 8 | *p*<0.001 | *p*<0.001 |
| *ras2∆* | 38.9 | 592 | 59.0 | 540 | 5 | *p*<0.001 | *p*<0.001 |
| *ras2*Δ *sch9*Δ | 63.0 | 969 | n/a | n/a | 4 | *p*<0.001 | *p*<0.001 |
| *ras2*Δ *sch9*Δ *rim15*Δ | 49.6 | 763 | 59.6 | 545 | 3 | *p*<0.001 | *p*<0.001 |
| ***Glucose reduction (SC+0.5% glucose)*** | | | | | | | |
| WT (DBY746) | 31.8 | 489 | n/a | n/a | 5 |  | *p*<0.001 |
| *msn2/4∆* | 16.0 | 246 | 28.3 | 259 | 5 | *p*<0.001 | *p*<0.001 |
| *gis1∆* | 16.2 | 249 | 29.3 | 268 | 4 | *p*<0.001 | *p*<0.001 |
| *msn2/4∆ gis1∆* | 10.3 | 158 | 16.1 | 147 | 4 | *p*<0.001 | *p*<0.01 |
| *rim15∆* | 5.7 | 88 | 11.0 | 101 | 5 | *p*<0.001 |  |

^1^ Data presented were calculated from pair matched, pooled experiments. ^2^ Percent of wild type (DBY746) in SDC. ^3^ “n” indicates the number of cultures analyzed. n/a, data not available. ^4^ *p*-value for mean CLS of mutants compared to that of wild type under CR, ANOVA, Tukey's Multiple Comparison Test, ^5^ *p*-value for mean CLS of wild type or mutants under CR *vs.* wild type in SDC, ANOVA, Tukey's Multiple Comparison Test.
